# "The Hospital" Nursing Mirror

**Published:** 1898-12-03

**Authors:** 


### "The Hospital" Nursing Mirror.

Being the Nursing Section of "The Hospital."

[Contributions for this Section of "The Hospital" should be addressed to the Editor, The Hospital, 28 & 29, Southampton Street, Strand, London, W.C., and should have the word "Nursing" plainly written in left-hand top corner of the envelope.]

### News from the Nursing World.

### MIDDLESEX NEEDLEWORK GUILD.

The Queen, for the fourth year in succession, has permitted the Banqueting Chamber, Ante-room, and Armoury of St. James's Palace to be used for the exhibition of garments made for charitable purposes by the Middlesex Needlework Guild prior to distribution. The Duchess of Albany is the president, and has been actively engaged in the preparations during the past week. The number of garments this year is 23,445, and they are of excellent work and quality. Several of the Royal Family take an interest in the display, which took place on November 30th. The President urged upon the presidents of groups the importance of keeping a store of garments ready for distribution, as in cases of emergency, like the epidemic at Maidstone, such a reserve proves most valuable.

### HONOUR FOR AN ENGLISH NURSE.

English nurses cannot fail to rejoice that one of their number had the privilege of rendering the last services to the ill-fated Empress of Austria, whose dastardly murder aroused the indignation of Europe. As it happened, Sister Caroline Robinson, one of the nurses attached to the Hollond Institute for the last four years, was in attendance on a patient at the Hotel Beau Rivage, Geneva, and, upon hearing of the terrible calamity, immediately proffered her services to the Lady-in-Waiting, which were gratefully accepted. The Emperor of Austria has just sent her an Order of the Second Class of the Grand Cross for Women and a certificate declaring the reason for which the decoration was bestowed.

### FOR THE NURSES OF ARGYLL.

The Lord Provost of Glasgow opened the bazaar in St. Andrew's Hall, in the absence of the Duke of Argyll through indisposition. There were present on the platform the Duchess of Argyll, Sir James King, Bart., Sir John Muir, Bart., and Admiral Sir Fitzroy Maclean, Bart. The bazaar is in aid of the funds of the Argyllshire Nursing Association, a society very near the Duchess of Argyll's heart, and she has taken much thought in the preparation and arrangements in order to assure its success. Her Grace's own stall displayed a great variety of tempting items at equally varied prices, amongst which were a couple of paintings by the Duke. Mr. John Muir, of Newcastle-on-Tyne, designed this stall, as well as the decorations of the halls. The novel feature was the introduction of four Iona crosses of grey stone. Sèvres ware, Dutch pottery, Japanese dolls, and Armenian pottery were to be found on the Kintyre Stall next to that of Argyll. Lady Campbell, of Blythswood, and the Countess of Glasgow superintended the Glasgow Stall. The Mid-Argyllshire Stall was very effective, and the articles upon it were the most valuable in the bazaar. Indian work, oak carving, and cushions were plentiful. Fur rugs, fans, and nick-nacks occupied the Maclean Stall, which was draped with the tartan of the clan. The Lorne Stall was characterised by the multitudinous number and variety of its exhibits. Amongst the amusements not the least attractive was the exhibition of baby lions, leopards, and a young bear lent for the occasion from the Zoo. The first day's takings amounted to £2,256.

### ROYAL FREE HOSPITAL.

A meeting was held on the 22nd inst. at the Royal Free Hospital to discuss a memorial for the late curator of the museum, Miss Mabel Webb, M.B. The chairman of the committee, Mr. Justice Bruce, presided, and Dr. R. H. P. Crawfurd proposed the following resolution: "That the memorial should take the form of some improvement in the pathological department of the hospital," and explained that the scheme embraced the rebuilding of the museum, with additional provision for the advancement of pathological work. The cost is estimated at £1,500, and Mrs. Webb has undertaken to bear the greater part of the outlay. Subscriptions amounting to £200 have already been received, and the Secretary of the Royal Free Hospital will be glad to receive further donations for this purpose.

### CHANGE OF MATRONS.

Miss Gould, who has been matron of the Sydney Hospital, N.S.W., for the last seven and a half years, has resigned her work there upon her appointment to the hospital at Rydalmere. On October 17th, 1898, the board-room at the Sydney Hospital was prettily decorated and thronged with guests, to witness the presentation to her of many valuable souvenirs of the kindly appreciation and personal affection in which she is held. Dr. Thomas Evans presided, and gave her a silver salver, on behalf of the honorary medical staff; Dr. M'Clelland a silver tea service, for the resident medical staff; and Mr. Jones a marble clock, for the male staff. Sister Creal, who has been trained by Miss Gould, and has been appointed acting matron, presented a travelling bag, a lounge, and various useful articles, on behalf of the nursing staff.

### A TIRESOME RESTRICTION.

How can a house stand when divided against itself—especially a workhouse? Amidst the many perplexities that harass the mind and conscience of the thoughtful Guardian, it is a little too bad to have one's fellows disloyal to the constitution under which they all work, and to which, sooner or later, they must all yield obedience. It is this sort of thing that lies at the root of the troubles at Yarmouth, and its vexatious results are not inaptly illustrated by the manner in which the nurses receive medical advice. By a majority of two, the Board lately refused to grant the medical officer a reasonable fee for attending the nurses, not that any Guardian is unwilling to provide necessary medical care, but because the service must be paid for in their own way. And their own way is unique. When a nurse has been under medical treatment, the Guardians inquire into the case, and, if they think it a fit and proper one, they present the nurse with a gratuity with which to pay the bill!

### AMATEUR THEORISTS.

The announcement that the course of instruction for male and female nurses provided by the Departmental School at the St. Anne Clinic, Paris, is open to the general public must have an odd sound to the ears of English nurses, and few amongst them would relish the thought of a similar proceeding on this side of the Channel. Whilst practical dexterity is often useful, a little technical knowledge is a somewhat dangerous commodity, therefore it is a pretty safe rule to learn to do many things as well as possible, and to keep the technical instruction abreast of the performance, for the knowing how to do a thing and the doing of it are very different affairs. The terrors of sick rooms are not lessened to the imagination by the prospect of each member of the community being able to give advice as to the nursing of every case under the sun, in the same happy manner in which he or she is now prone to prescribe its medical treatment. This little peculiarity has afforded the professional jester many a butt at which to hurl his wit, and the errors of the amateur theorists in nursing cannot fail to open an equally wide field either for amusement or dismay.

### A GENEROUS GIFT TO GATESHEAD.

Mr. Walter Willson, president of the Gateshead Nursing Association, has offered to build a new home for the nurses attached to it. A special meeting was called, at which the Rector of Gateshead took the chair, and the Secretary of the Association had the pleasure of reading a letter from Mr. Willson announcing his intentions in the matter. He has already secured a site in Coatsworth Road, and the plans of the new structure accompanied his letter. The property will be given to the town and vested in trustees for the use of the association, with the sole proviso that in the event of its failure, owing to inadequate support, the house should only be used as a private dwelling. Mr. Willson took much interest in the work of the nurses during the year of his mayoralty, and his gift is the result of his appreciation of its value.

### A STUFF FROCK.

A nurse in the employ of the Barnsley Board of Guardians has recently tendered her resignation for the ostensible reason that she was compelled to wear one stuff dress on all occasions. She had represented to the matron that she should prefer one of washing material, but was informed that "She was in bondage, and that she must either wear the dress or resign." At least, this is her version of the affair, and although there is another which points a different way, the fact elicited by the inquiry was that the night superintendent at this workhouse has only one stuff dress provided in which to attend all cases—maternity, dirty, and laying out of the dead. It is difficult to conceive a nastier idea, or one less in accordance with hygienic regulations. We trust that this rule is peculiar to Barnsley, and that the next night nurse will be well supplied with delightfully clean washing frocks.

### SCOTTISH NEEDLEWORK GUILD.

In connection with the Scottish branch of Queen Victoria's Jubilee Institute for Nurses is a Needlework Guild, which provides the necessitous sick in the care of the "Queen's" nurses with clothing. The Countess of Galloway is president for the Wigtownshire district, and recently inspected the 214 garments which are the fruits of this year's work. A sum of £7 8s. 6d. was also collected, and, after the local district nurse had made her selection, the remainder was sent to the headquarters in Edinburgh.

### ERITH HOUSE, TORQUAY.

How many sick ladies are there vegetating in uncongenial rooms through the foggy months of an English winter to whom the thought of a few months at Torquay would suggest unattainable comfort, and yet there are many homes ready to receive them. Our attention has once more been called to Erith House, Torquay, by a correspondent, who speaks most highly of the food and comfort, whilst we see that the terms are moderate and inclusive. Two ladies may share a room upon the payment of 17s. 6d. per week, and the luxury of a separate room may be enjoyed from 20s. to 22s. Those for whom the home was established are the widows and daughters of officers and clergymen, governesses, and members of professional classes. The patroness is the Duchess of York, and the treasurer and secretary is Mr. R. J. Parr. We have noted the home from its helpful side. It also affords such as are willing an opportunity of helping delicate gentlewomen in a very efficient way, and subscriptions to enable the work to be carried on are much needed.

### SHORT ITEMS.

By an oversight the address of the Nurses' Co-operation was given last week as 8, Old Cavendish Street, instead of 8, New Cavendish Street. We trust none of our readers have been inconvenienced by the error.—The lady superintendent of the Barry Nursing Association (Miss Sykes) informs us that the report of her resignation is unfounded.—The Local Government Board have sanctioned the Wrexham Guardians' grant of £5 a year to the funds of Brymbo District Jubilee Nursing Association.—At a large and influential meeting held at Torres, Aberdeen, it was unanimously resolved to form a nursing association for the free nursing of the poor. Several annual subscriptions have already been promised.—A chapel in connection with the Maternity Charity and Nurses' Home at Plaistow was opened for Divine service on Saturday last by the Bishop of Colchester. The building is a part of an old grammar school, belonging in days gone by to a vicar of St. Mary's. The freehold building was purchased and presented to the home last year by a lady, and the nurses past and present have subscribed to enlarge it and prepare it for its present use. It is dedicated to St. Veronica.—Sir Squire Bancroft is as ever active in the cause of charity. He will give his reading of Charles Dickens' "Christmas Carol," on December 1st, 1898, in aid of the North London Nursing Association, in Highbury Athenæum.—On the afternoon of Saturday, November 26th, the children at the Incurable Hospital, 2, Maida Vale, W., gave a musical fairy play in aid of the Universities' Mission to Central Africa, in which they are interested. The Lady Superintendent (Mrs. Bruce) arranged the music and the words of one of the songs, and the entertainment was most successful, and wonderful considering the helpless condition of many of the children.

### Nursing in Diseases of the Throat, Nose, and Ear.

By MACLEOD YEARSLEY, F.R.C.S.Eng., Assistant Surgeon to the Royal Ear Hospital, Surgeon-in-Charge of the Department for Diseases of the Throat, Nose, and Ear, the Farringdon General Dispensary, Hon. Surgeon for Diseases of the Throat and Ear, the Governesses' Home, &c.

### VIII.—AFTER-TREATMENT OF OPERATIONS ON THE EAR.

IN our fourth article the various operations upon the ear were dealt with, and in the present paper their after-treatment has to be dealt with. It is, however, somewhat difficult to know exactly how much to say on this subject, as in different institutions and with different surgeons the amount of work left to the nurse varies much. One thing cannot be too carefully kept in view however great or however little the work may be, and that is the paramount necessity of perfect asepsis or antisepsis in the after-treatment of operations on the ear. Much of the success of the work which has been done will depend upon the proper observance of this rule, and, whether the surgeon or the house surgeon dresses the case himself, or whether the nurse is entrusted therewith, the latter must never relax her vigilance as regards the instruments and solutions used, the dressings required, or, last but not least, the condition of her own hands.

And here, although perhaps not strictly within the limits of these articles, it will not be amiss to devote a few words to the cleansing of the hands. They should be well washed with an etherial soap, or with ordinary soap preceded by the application of ammonia or ether, and not only washed but scrubbed well with the nail brush back, front, knuckles, and nails, the washing extending above the wrists. They should then be rinsed in clean water, dried on a clean towel, and dipped in some antiseptic such as 1 in 40 carbolic lotion. Once cleansed and antisepticised, such hands must not be used for touching anything which has not been made surgically clean. It need scarcely be said that this applies only to the nurse who may have to help in an operation (touching and handing instruments, &c.), or apply a dressing. All instruments used in the after treatment of a case should be sterilised by boiling and placed in antiseptic.

Keeping to the operations discussed in Article IV., the after treatment of such small manipulations as the removal of polypi and granulations, paracentesis of the membrana tympani, and the opening of aural furuncles is, as a rule, left to the patient at home. After the removal of polypi or the curetting of granulations the ear must be gently syringed with a hot antiseptic lotion and lightly packed with a strip of gauze, and after any such operation upon the ear it cannot be too firmly impressed upon the reader that no patient should be allowed to leave the institution without an antiseptic dressing in the meatus. The author has known neglect of this precaution lead to very untoward results.

After paracentesis of the tympanic membrane the meatus should be lightly plugged as directed. Otologists vary in their opinions as to the after treatment of this little operation. Many (the author amongst them) prefer simply to pack with gauze and not syringe the ear at all. Syringing should certainly not be needed if the ear has been properly purified before the operation.

The importance of antisepsis after the incision of boils in the ear cannot be too strongly insisted upon. It has been recommended to anoint the boil after incision with carbolic glycerine or some other antiseptic to prevent bacteria from invading neighbouring follicles. Swabbing the meatus with Lister's "strong mixture" (see Article IV.) certainly gives good results. Should the nurse be in charge of a patient suffering with aural furunculosis she must see that the treatment after their incision is carefully and punctually carried out. The writer always follows up such incisions by instillations of spirit. In spite of the often intense irritation which follows aural furunculosis the patient must on no account be allowed to scratch the ear.

Passing now to the more serious operations, the first to be dealt with is the excision of the ossicles. After this procedure the ear is thoroughly anti-septicised with hot sublimat (1 in 2,000) or carbolic (1 in 20) solution, and carefully plugged with antiseptic gauze. The gauze usually used is soaked in 1 in 40 carbolic lotion,

However the ear has been dressed, it should not be touched until the surgeon has seen it, unless he has given orders to the contrary. The patient may require confinement to his bed for one or two days after the operation on account of vertigo, but this dizziness varies much and can generally be relieved by appropriate treatment. Pain in the ear following the operation should be treated by placing hot, dry wool or flannels over the side of the head.

After the removal of exostoses little more need be said; the meatus is plugged with gauze, and this plugging usually requires continuing longer than in operations on the middle ear, in order to prevent contraction of the canal. From one to three weeks is usually sufficient, but the reason just given may require its longer continuance. If the removal of the growth has required the turning forward of the auricle, the incision will have been sutured, and the wound will merely require ordinary treatment.

MASTOID OPERATIONS.—Every case of mastoid operation varies more or less in its after treatment. The average duration of the wound treatment until complete cicatrisation varies in the regular course from two to five weeks, but some cases require longer than this. It may at once be said that the treatment after opening the mastoid antrum is of great importance for the final result. The necessity for the most careful antiseptics and most stringent supervision cannot be too strongly insisted upon. In some cases it may be sufficient to change the dressing every three or four days, but the writer prefers to do the first dressing in the first twenty-four hours after the operation. As a general rule this dressing consists of the removal of the gauze, plugging the meatus, and gentle irrigation with 1 in 40 carbolic lotion, followed by replugging. A drainage tube is rarely required, and the wound behind the ear is generally sutured and allowed to heal by first ntention. Unless there is any reason to the contrary, the after dressings need be done only every two or three days, the indications for a daily change being (1) return of pain, (2) increase of temperature, (3) rapid soaking of the bandage, (4) continuation of septic suppuration.

Should the nurse be entrusted with the dressings, she must keep ever before her the absolute necessity for strict antisepsis. Should the bandages, gauze, or gauze plug stick, they must not be roughly or forcibly removed, but soaked with hot antiseptic lotion, and a little care and trouble should be exercised in their removal.

The after-treatment of operations for otitic, cerebral, and cerebellar abscesses and for septic thrombosis of the lateral sinus will remain in the hands of the surgeon, the nurse acting merely as an accessory, although an accessory of considerable importance. The necessity for the strictest antisepsis is, if anything, even more to be insisted upon than before. The management of cerebral cases belongs more to the domain of general surgery, and as the nurse will probably have already received practical training in the subject more than this passing reference thereto need not be made. After the removal of tumours from the auricle, the treatment will not differ from that of any other operation wound elsewhere.

### Training in the Provinces.

(*Continued from page* 86.)

### ROYAL PORTSMOUTH HOSPITAL (133 Beds).

### Terms of Training.

Candidates for training at the Royal Portsmouth Hospital should be not less than 22 years of age. If accepted after the month's trial the probationer enters upon a term of three years' training in the wards. All probationers pay an entrance fee of £1 1s.; salary begins at once at £8 per annum, rising to £14 for the second year, and to £20 for the third. On the recommendation of the matron they are entitled to a "crown of merit" after two years' approved service, receiving an extra £2 per annum. Private nurses' salaries range from £32 to £36 per annum, and sisters are paid £30 per annum. Laundry and indoor uniform (three print dresses, three caps, six aprons and pairs of sleeves, six collars, and when private nursing one more print dress) are provided by the hospital. Private nurses must now be fully trained and certificated, no nurse being sent out until she has completed her three years' training.

A certain number of special probationers are received for one year's training on payment of a premium of £31 10s. As regards work, they are on exactly the same footing as the paid probationers, and if at the end of the year they wish to continue their training they can do so, with the consent of the committee, on the same terms as the ordinary probationers.

A three years' certificate from a recognised training school is a necessary qualification for promotion to the charge of wards. Night duty is taken by the probationers for not more than six months consecutively, in rotation with day duty. Lectures are given by the resident staff, and examinations are held, certificates being granted to those nurses who satisfactorily complete their engagement.

### Hours On and Off Duty.

Probationers are on duty in the wards between the hours of 7 a.m. and 8 p.m., with two hours off duty daily, extended to five hours once a week, and time for meals. Sisters are on duty from 8 a.m. to 8 45 p.m., with two hours daily for recreation, and five hours once a week. On Sundays the hours off duty are from 10 a.m. to 1 p.m., or from 4 to 9 p.m. alternately. Three weeks' leave is allowed annually. Night nurses are on duty from 8.30 p.m. to 8 a.m. The sisters and nurses on day duty have one whole day off duty once a month; by arranging with the assistant matron the evening before, breakfast is sent to their rooms, or if an evening pass can be arranged they may, by permission of the matron, leave the hospital for the night, returning at 9 p.m. the following day.

### Meals.

All meals except the sisters' lunch and tea and the night nurses' midnight meal are served in the dining-rooms. Breakfast for the day staff is at 6.30 a.m. for the probationers and an hour later for sisters; lunch is served from 9 to 10 a.m., dinners at 1 and 1.30 p.m., tea from 4 to 5 p.m., and supper at 8.45 p.m.

### SOUTH DEVON AND EAST CORNWALL HOSPITAL, PLYMOUTH (130 Beds).

### Terms of Training.

Before being accepted as a non-paying probationer at the South Devon and East Cornwall Hospital a three months' trial is required of candidates, who should be between 23 and 30 years of age. The course of training is for three years, and examinations are held at the end of the first and second years. A certificate is given on satisfactorily completing the engagement. Fees of £1 1s. per week are paid by probationers for the first six months; the second year payment begins at £15, and is increased to £18 for the third year. Sisters' salaries range from £30 to £35 per annum. (The wards are small, the largest containing 27 beds, and most of the rest only 18.) Laundry and indoor uniform are provided by the hospital for all members of the regular hospital staff. Private nurses only have out-of-door uniform. Lectures are given by the visiting staff and by the matron to the nurses during their training, and classes are held by the sisters. Probationers are expected to pass the examination at the end of the second year before they are placed on staff duty during the third year.

Six vacancies are reserved for paying probationers, who are received for a year's training with the idea of affording an opportunity of gaining some experience in nursing to those who intend entering a London training school later on. For the first six months they pay a premium of £1 1s. per week, and they provide their own laundry and uniform. No certificates are granted to these short-service probationers unless they remain in the hospital for the full term of three years. For promotion to the post of sister three years' training is an essential qualification, and one year on the staff. Night duty is taken by the nurses in rotation with day duty, usually for three months at a time.

### Hours On and Off Duty.

Probationers and staff nurses' hours on duty are between 7.20 a.m. and 9 p.m.; sisters go on duty ten minutes later in the morning. Two and a half hours are allowed daily for recreation, exclusive of meal times. A half day, from 2 to 10, is given fortnightly, and on alternate Sundays leave is given from 3.30 to 9.30, or from 10 a.m. to 1.30 p.m. The time table is elastic, as is often the case in country hospitals, and many pleasant excursions are made for the nurses during the summer months when work may happen to be light. Four weeks' holiday are allowed annually, and this is exclusive of sick leave. Nurses on the sick list are paid full salary for three months, and if summoned to nurse their nearest relatives receive half pay during their absence from duty.

### Meals.

All meals, with the exception of the night nurses' midnight tea, are served in the dining rooms, no "allowances" being given to day nurses. Stores for the night nurses are sent to the wards, where a special safe is provided for their keeping. Special attention is paid to the dietary at the South Devon Hospital, where it has been proved by experience that a varied menu costs no more than one of dull routine, and is infinitely more wholesome and acceptable. The hours for meals are as follows: Day nurses' breakfast, 7 a.m.; lunch, 10 a.m.; dinner, 12 30; tea, 4.30; supper, 9 p.m. Sisters breakfast at 8; lunch is at 10.30; dinner at 1 30; tea, 4 to 5; and supper at 9 p.m.

### Where to Go.

Royal British Nurses' Association.—At 11, Chandos Street, Cavendish Square, W., the first Sessional Lecture of the season will be given on "Our Hospitals" by Miss Rosalind Pritchard. The lecture, which will be illustrated by an original and comprehensive series of limelight views, will take place on Friday, December 9th, at eight p.m., and a few seats will be available for the general public.

At the Empress Rooms, Royal Palace Hotel, Kensington, a ball will be given on December 9th, 1898, in aid of the funds of the Royal London Ophthalmic Hospital, Moorfields. Tickets price £1 1s. (17s. 6d. a piece for four), may be obtained from the Secretary at the hospital.

### Presentations.

Miss E. Rudd was presented with an illuminated address and a purse of gold on November 11th by the workmen of Redcar Iron Works and other residents of the district of Chatham and Warrenby.

### Nursing in Paris Hospitals.

### C.—THE NURSING SISTERS.

### VII.—Their Present Domain: The Boucicaut Hospital.

The Sisters have had a remarkable triumph this last winter in taking possession of the new model hospital bequeathed to the Assistance Publique by that remarkable Lady Bountiful of the Bon Marché Stores, the late Madame Boucicaut. Of course, it is no relenting spirit in the Municipal Council which has brought this about; but even their fanatical secularism could hardly refuse eight million francs because it came with the stipulation that the Sisters must be employed.

The foundation of the Boucicaut Hospital is likely to be such an important episode in the struggle between Sisters and laics that a sketch of its origin and an account of its installation are worth giving here. On October 8th, 1887, Madame Boucicaut died, leaving the Assistance Publique of Paris her residuary legatee. The Assistance was authorised to spend (after payment of all special legacies) the sum of two millions for general purposes. This done, if the residuary estate reached eight millions, the Assistance Publique was obliged to build and keep up a hospital in Paris. In case the residue should be less than eight millions, the administration was relieved of the necessity of building the hospital and might employ the funds for different charitable works, with the consent of the executors.

On January 1st, 1889, the legacy amounted to 7,500,000 francs. The administration therefore might have considered that they were not bound to build the hospital, but they considered they could not make a better use of the money. In accord with the executors they bought land situated between the Rues de Cévennes, Lourmel, Lacordaire, and de la Convention, at Javel in the southwest, near the Porte de Sèvres. They then prepared a scheme for the new hospital. This scheme, breaking away from all traditions, did not have as a basis the separation of the sexes, but, instead of this, the absolute separation of the medical and surgical services, the latter being sub-divided into infectious and non-infectious cases. Nor were the beds for the two sexes equally divided; the men were given one-third more beds, the demands for admission by men being always more numerous than those by women. The proposed erections were put up to competition in August, 1892. The jury consisted of 15 persons, viz., five members of the Municipal Council, two of the Conseil de Surveillance of the Assistance Publique, a doctor and a surgeon of the hospitals, one of Madame Boucicaut's executors, three architects named by the competitors, the Director of Works of the City of Paris, and the Director of the Assistance Publique. Forty-three plans were submitted, and the one chosen was that of MM. Legros and Son. Their plan was somewhat modified by the Supervising Council of the Assistance Publique on June 7th, 1894. It was approved by the Municipal Council on July 26th following, and on October 18th of the same year the work commenced. The hospital contains 152 beds, thus divided: Medicine, 72 beds, 44 for men and 28 for women; surgery, 60 beds, 36 for men and 23 for women; and lying-in, 20 beds. The extent of the grounds is 30,000 mètres, the buildings covering 7,500 mètres, the rest being courts and gardens. The wards extend from east to west, the patients thus having the sun at all hours of the day. The buildings, which above ground are separated from each other, are united by underground galleries. These galleries are lofty, wide, and lighted and ventilated by skylights in the gardens, having rails which enable all necessary transport in the different services. Lifts do away with the introduction of persons unconnected with the medical service into the portion reserved for the sick, and consequent danger of contagion.

The Sisters can boast of having charge of the best arranged and "up-to-date" hospital in Paris. The establishment consists of seven buildings, as follows: (1) A principal building, called the building of the administration, fronting the Rue de la Convention, having on the ground floor, to the right of the entrance, the offices of the administration, a medical consultation room (with separate hall for contagious cases), examination room, and a pharmacy; to the left the porter's lodge, surgical consultation room (with rooms for dressings), and dentistry consultation room; on the first floor are the apartments of the director, the chaplain, and rooms for house doctors, house surgeons, &c. (2) To the left of this building, some six mètres away, are two pavilions of one storey for observation, each containing four rooms for patients, a nurses' room, hall, and water-closet. (3) To the right of the central avenue, starting from the administrative building, are four medical pavilions, the first two for men, the others for women. Each pavilion contains a common ward, at the end of which are two isolation rooms, a day ward, which may be used as a refectory, a bath-room, pantry, a water-closet, a lavatory, and a linen-room. (4) On the left of the central avenue two groups of two pavilions, differing only from the medical pavilions in that the first and last pavilions have each an operating pavilion, with necessary adjuncts, joined to the patients' pavilions by closed galleries; in addition, each ward, medical and surgical, is furnished with a loggia, a large glazed space, forming a winter garden, where the patients can assemble in the daytime and amuse themselves by reading, permissible games, writing, or other occupations. (5) At the end of the Avenue is the Bon Marché Pavilion; in front of this pavilion there is a bust of Madame Boucicaut. (6) Behind the medical pavilions at the angle of the Rue Lacordaire and the Rue de Cévennes is the Maternité; Parallel with the Rue Lacordaire is a pavilion for the isolation of suspicious cases, with, on the ground floor, a pantry, a kitchen, two ante-rooms, and a room for the midwife; on the first floor the doctor's office, and two rooms with two beds each for patients, baths, and water-closets; behind, but separated by a court, with lawn and flowers, is the real lying-in hospital, a pavilion of two wings at right angles, one in the direction of the Rue de Cévennes, the other perpendicular with it; this pavilion has a special entrance in the Rue Lacordaire; on the ground floor is a vast waiting room, a consultation room, an examination room, a clothes-room, bath-room, doctors' room, pharmacy museum, a room for the midwives, another bath-room, water-closets, a dormitory with four beds for pregnant women, and six rooms for the staff; on first floor work-room, baths, operation-room, pantry, linen-room, water-closets, and lying-in ward of 16 beds. (7) General service: These (kitchen, linen-room, pharmacy, Sisters' quarters, staff dormitory) are united in a building behind the Bon Marché pavilion; in the Rue de Lourmel is the entrance to two large pavilions, on the ground floor of which are, on the right the laundry, the disinfecting chamber; and on the left, the mortuary and the laboratory.

All the pavilions, as I have already stated, are united to the general services by a subterranean gallery communicating with the ground floor by means of staircases and lifts. It is by this gallery that all the services of the establishment are carried out. The heating is by steam, and the lighting by electricity. This double service is carried out by underground engines, situated between the general service building and the Rue de Cévennes.

The administrative building is of stone, the others are of brick and iron, the roofs being of tiles. The wards are 9 mètres wide and 6½ mètres high, and the supply of air is not less than 80 cubic mètres per hour per patient. The verandahs are filled with foliage plants at the end of each ward, where the patients can rest during the day.

This last is a happy exception to the general bleak aspect of the Paris wards, as compared with those of London hospitals in the matter of cheerful accessories, especially of flowers. Altogether the new Boucicaut Hospital is likely to mark an era in the Paris administration, and its success will, of course, redound in a measure to the credit of the Sisters in charge.

EDMOND R. SPEARMAN.

### The Strangers' Hospital, Rio de Janeiro.

THE Strangers' Hospital, Rio de Janeiro, is rapidly becoming a bye-word for general misgovernment. It may be interesting briefly to recount the history of this institution. Some ten years ago Mr. Lamoureux, editor and proprietor of the *Rio News*, originated a scheme for the establishment of an English hospital in Rio. He succeeded in course of time in raising with the help of residents in Rio the sum necessary for its erection, and in 1893 the hospital was opened. Since that time up to the present moment the history of the Strangers' Hospital has been one of continued internal dissatisfaction and change. During the six years of its existence there have been no less than three matrons and eighteen nurses (the staff numbering four) of whom but two have completed their contracts, not counting the four who went out from England in September of this year. There have been during the past two and a half years two entire changes of staff, the nurses resigning in a body in the first instance, one of their number having been dismissed without an enquiry into the cause; the second general resignation, which has taken place this autumn, being due to difficulties with the Matron, Miss Jackson, the directors entirely refusing to listen to the nurses' complaints. Four nurses have just gone out to fill the vacancies thus created, and those who have intimate knowledge of the circumstances which have led to past troubles await further developments with interest.

The changes of staff during these years work out in this manner, according to the statement of Mr. Lamoureux, who has all through taken much interest in the welfare of the hospital, in the *Rio News*. "Of three matrons and eighteen nurses engaged during these six years, one matron and two nurses were engaged here (Rio), two nurses were brought up from the River Plate, and two matrons and fourteen nurses were brought out from England, the latter at an expense, say, of £32 each. The total expense has therefore been £464 in travelling expenses and outfits. This shows that the hospital has engaged an average of three nurses a year at an expense of about £77. . . ."

Leaving past history, let us come to the reasons for the most recent change in the staff. Most serious charges are made against Miss Jackson by various writers in the *Rio News*, charges of carelessness in matters of disinfection, of personal unkindness, &c., and Miss Ginns, a member of the late staff, who has been appointed Matron of the São Paulo Hospital, writes as follows in the issue of that paper of September 27th last:—

"Hospital Isolamento, São Paulo, September 22nd.

"DEAR SIR,—As one of the last staff of nurses at the Strangers' Hospital, allow me to state we did not suffer from the overkindness of the matron, unless daily and hourly petty tyranny could be called by that name. A paper signed by the whole nursing staff was sent in to the board of directors of the hospital in March which demanded a full enquiry. It was simply ignored, although it would have had full investigation at the hands of any hospital board in England. It would be interesting to the subscribers if they saw for themselves the nature of the complaints we made. I had the post of matron offered to me the last week in March at the Hospital Isolamento, São Paulo, and it was April 14th before I sent in my resignation, five days after the board meeting when it was decided to ignore our complaints, and I should not have done so if we had received justice at the hands of the directors, for I had many reasons for wishing to stay in Rio.—Faithfully yours,

"ALICE M. GINNS."

During Miss Jackson's administration, covering something under three years, she has had ten nurses on her staff, of whom one died and the rest declared themselves unable to complete their contract under her. Yet in spite of all this, in spite of the remonstrances of subscribers, the directors have continued to ignore the whole matter. A general meeting of subscribers was held in October, at which this non-intervention policy was upheld and supported by the majority of those present, Mr. Lamoureux's amendment, rejecting that part of the report dealing with the action of the nurses, being negatived. The directors have therefore resolved to make no inquiry into the formal complaints lodged by one nurse after another and by the whole late staff conjointly, and the supporters of the hospital have acquiesced in this manifestly unjust proceeding. Without entering into the merits or demerits of the internal management of the Strangers' Hospital, there is but one view of the matter to be taken. No improvement in the present anarchic condition of things can possibly be anticipated until a thorough and complete public investigation has been made into the administration of the hospital.

### Appointments.

WORKHOUSE INFIRMARY, CHELTENHAM. — Miss Jane Jeffrey has been appointed Superintendent Nurse of this institution. She was trained at the Royal Edinburgh Hospital for Sick Children, at the Middlesex Hospital, and at the Clapham Maternity Hospital. Miss Jeffrey holds the L.O.S., and has been superintendent nurse at Newton Abbot Workhouse Infirmary, and the Infirmary, Warrington.

ROYAL ALBERT HOSPITAL, DEVONPORT.—Miss Annie Glover, who has been successively sister of the children's wards and theatre and senior sister of this hospital, has been appointed Matron. Miss Glover was trained at King's College Hospital, London.

ST. GILES' INFIRMARY, CAMBERWELL.—Miss Cotterell was appointed on November 23rd, 1898, Superintendent of Nurses at this infirmary. She had previously been night superintendent at the Brook Hospital.

### A Book and its Story.

"LIFE IS LIFE."

This little story* of Zack's "Life is Life" has a great charm about it, for not only is the plot attractive, but the telling of it is both artistic and powerful. There is no doubt about it that the authoress is gifted in no small degree, and that, besides having the pen of a ready writer, she has great powers of imagination, combined with a highly observant mind. Now and again, just here and there, among the tales and episodes comprising this book, one comes upon trifling evidences that the writer is perhaps wanting in that perfection of detail which marks the mature writer, but that Zack has a literary future before her there is no doubt whatsoever, and one trusts for the sake of the reading public that she will carry out the high ambitions one forms for such a delightful pen as hers.

First among the collection of stories and episodes comes the longest one, after which Zack's volume is named—"Life is Life"—and to this one alone we will confine our attention now. The plot opens with a description of an old English home: "The old Jacobean house, visible from a bend in the avenue, had an air of fallen fortunes; across the sleepy alleys grass crept undisturbed, and in the old-world gardens old-world flowers stretched up, cramped and cold, to the gaze of the October sun." Here in the opening chapter, within sight of Thursby Grange above described, we are introduced to a young boy who, in after years, plays the chief part in the story, and to a gamekeeper, by name Wilkie. Wilkie is a character of no importance in the subsequent development of the plot, but it is he who here in the first few pages gives us the clue to Master Humphrey's identity, around which, even to the boy himself, a deep mystery had hung; this mystery the boy is keenly anxious to have removed, and he urges his companion, the old poacher-gamekeeper, to enlighten him, and, in his own manner, Wilkie does so. He had been present at Thursby Grange when, for the first time, the boy had made his appearance. At length he describes the scene, urged by the impatient eagerness of his importunate listener.

"Wull," said Wilkie—he was describing the events of a certain memorable night at Thursby Grange when he was called upon to see the master—"I hadn't much more than laid out my tongue for the next wud when Henschel coomed in to say as how Dick Atter, he as wor the Cap'en's man, wanted to speak to the Squire most uncommon particular. I saw his honour turn a bit whitish. 'Let him coome in,' he says, windervul unconsarned, an' in Dick coomed accordin'. He wor karryin' a quare dumped up sorter skiddik; but he brought up his right hand to his face, military fashion, turrible respactful. Dick he tooked a packet o' summat from his coat pocket; 'twor tied this way an' thic, and sealed most all over. 'The Cap'en said I wor to give 'ee this, sir,' he said. After a bit, what shud tummil out but the Cap'en's gold watch and chain, an' a ring he used to wear on the little finger of his left hand! The Squire he guved a great start, an' his face went reglar chalk-white. 'Where is your master?' he axed, sharp like. 'Dead, sir,' said Dick, and the poacher went on to explain how, at this news, the Squire walked to the window and stood and stared through the trees at the Black Swan Lake. After a while he turned round—'There was no message—nothing?' he asked. Dick put the big bundle down on a chair, and after unwinding o' stuff, what should plump out but yer honour's self, a little snip of a chile o' two years old. 'The Cap'en said I was to tell 'ee, sir, that he be a Thursby an' a gentleman.'"

More than this the boy could not gain from the old poacher. Anyhow, it was more than he had ever succeeded in eliciting from anyone among his surroundings at the Old House. But Wilkie, when questioned further as to who the boy's mother could be, was silent and could not say; and it was in these mysteries, deep and impenetrable, that the boy was brought up.

One day, some four years later, we find the Squire and his grandson seated in friendly converse in the great dining hall at Thursby Grange. "Humphrey was seated opposite the Squire, over his wine; the further end of the great dining hall was lost in shadow, against which the lights from the candelabra beat vainly . . . on the walls hung the portraits of Thursbys dead—all but the eyes, which, ever alert, peered down upon the boy. Humphrey glanced at the Squire sipping his wine, and wondered what were his thoughts; was he, too, haunted by those ever-vigilant eyes, or had he grown indifferent with years?" And then the two fell to talking; it was the last time they would dine together for many a day, as Humphrey was about to put into execution a long-planned project—to seek for further evidence as to his birth, or, as he put it to his grandfather, "to find that fellow Atter and sift his story to the bottom," and, despite all colder reasoning on his grandfather's part, the boy holds to his point, and determines to follow Atter to Australia, to which country, he is known to have returned. The truth as to this story meant a great deal to Humphrey, so he declared to the grandfather, who replied, "My lad, Atter's story was as impossible to prove as disprove." "You acknowledged me on slight evidence," said the boy. "And have not regretted it so far," the Squire answered. "I feel a Thursby, sir," the other added after a pause, "every bit of me." And, again later, the Squire remarked, "I can't help thinking you would be wiser to let the affair drop altogether," but the boy returned quickly, "I can't, sir. I would if it weren't for my mother; but—but you see she might be alive." The following day Humphrey left for Australia, and of his adventurous career there we must refer our readers to Zack's own account, which is by no means the least interesting part of the book, for here it is that the drama really develops.

To the boy Humphrey knowledge came at last, the knowledge for which he had sacrificed so much; it came at last, but it came as it comes to many of us, at a cost which is hard to pay: this is the "motif" of the whole story, and the subject is treated, despite its haunting pathos, with an artistic restraint on the writer's part which brings it home to her readers all the more forcibly. Humphrey's wanderings in quest of the truth led him among many thorny paths; but one ray of happiness was his, when he casts in his lot with some friendly souls, and finds in their unpretentious home a haven of refuge; it is here where the last scene in the volume is placed—on the eve of the boy's return to his English home. "Humphrey put up his hands, and felt the woman's face. 'Mother,' he said; 'you're crying!' 'None such thing,' she replied, indignantly. 'If I go home I shall come back again to you, I shall, I swear it.' 'Now jest you leave swearing alone; I ain't no friend to rash promises.' 'I don't believe you care for me, after all,' he said, in a hurt voice. 'You are terrible much of a chile, lad,' she answered, bending and kissing him. 'If I leave you, tell me something better than "life's life,"' he said, drawing her face close to his own. 'Ah, lad,' she answered; 'when a thing is, what does us gain by saying it isn't?' 'But it's a dreary philosophy,' he protested. 'What do the ills of life matter if us faces 'em courageous?' she answered; but her old, tired voice trembled, for of life and life's ills she was somewhat weary. Again he drew her face down towards his own. 'Mother, he asked, 'did you say life's life when first you knew Joe loved you?' 'Ay, on my knees I said it.' 'God bless you for having lived!' cried the boy. 'Oh, lad, lad,' she answered; 'I was never for denying the Almighty was the Almighty.'"

* "Life is Life." Zack. (London: Blackwood and Sons. 1898.)

### Everybody's Opinion.

[Correspondence on all subjects is invited, but we cannot in any way be responsible for the opinions expressed by our correspondents. No communication can be entertained if the name and address of the correspondent is not given, as a guarantee of good faith but not necessarily for publication, or unless one side of the paper only is written on.]

### DISTRICT NURSES.—THE DIFFICULTY OF GETTING AND RETAINING THEM.

"F. R. A." writes: A few remarks from a nurse's point of view may explain the long-felt difficulty of keeping well-trained nurses in needy districts. In going to a country district a nurse is expected to be the soul of perfection, and must make it her first duty to please the doctors, committee, and patients. When capable and well trained she may accomplish this, but it will probably be a work of time. If committees and people of the district would only give the nurse who is sent into their midst a little more consideration, how different her life would be. There would not then be so many nurses complaining of loneliness and of wishing to resign. Let it be remembered that many of them came from good homes or from a hospital where they have had many friends. To attain to their present position they have had training to go through and examinations to pass which must mark them as intelligent women, and not mere machines or paid agents. Have those ladies of the district who are well pleased to find that nurse is so valued by the poor ever considered how much they might do to make her life happy? Have they asked themselves how the nurse's time off duty is spent? Have they tried by little acts of kindness to show real sympathy for one who is willingly offering the best years of her life to the noble work of relieving the sufferings of humanity? Let members of churches in the various parishes who seek to follow their Master's example remember and befriend the nurses who labour so unceasingly to bring health to the sick and comfort to the dying. How often is a nurse called after a hard day's work to go forth at night to some poor sufferer, to whom she ministers with sweetness and patient self-denial? There are many who could with but little trouble do much to lighten her burden, strewing sunshine and light in her pathway.

### POOR LAW NURSE.

"Experienced" writes: With regard to nursing in workhouses, "An Infirmary Nurse" has hit upon the means of settling the vexed question when she states that house and infirmary should be entirely separately managed. A matron may be enlightened and very excellent in other matters, but if she dictates to, and encroaches upon the rights of a trained nurse, while lacking the knowledge of such herself, she must submit to be considered ignorant. I have had to deal with one who would turn ill at sight of a very sick patient or fly in disgust from an ugly sore, but who was frequently prepared to suggest her views and find fault with the nurse's method of treating the sick. She complained to me of one whom I knew to be a careful and reliable nurse because, according to her idea, she made her patient worse by changing soiled sheets for clean ones. When I went on duty there first I discovered there were but two towels in a ward of sixteen beds. Amongst the patients were two suffering from scabies, who had, in common with the others, to use these towels. When I pointed out the risk the majority of the patients ran who were obliged to use them, and asked for an increased supply, she told me the rules of that place existed for forty years, and that I would not be allowed to change them. And this was no "rude" and "ignorant" matron as I understand the phrase. I agree with "Infirmary Nurse"—the person best qualified to hold the post of workhouse matron is a trained nurse.

"A Surrey Guardian" writes: "A Workhouse Nurse," whose tale of woe appeared in your issue of November 5th, must have had her lines cast in very unpleasant places. Much that she says is true of many workhouses to-day—it was undoubtedly altogether true of the vast majority of workhouses fifteen or twenty years ago. The picture your correspondent draws is altogether of the old *régime*, when the quality of the nurses was almost as bad as that of the master and matron, the doctor and the guardians. A broad retrospect of the whole question, however, suggests to me that during late years there has been a general advance in all departments. The operation of the 1894 Local Government Act has revolutionised the constitution of boards of guardians, and with that change has come a transformation in every branch of workhouse administration. Even admitting the hampering effect of the Poor Law orders, there is still ample scope within their well-defined limits for progressive reform, and men and women in love with the work have not been slow to avail themselves of all the chances offered. Speaking in the light of my own experience, I can say that the 1894 Act lifted the dead hand from the board with which I am connected, and that one of the first things the new guardians devoted themselves to was the placing of the nursing staff on a basis satisfactory alike to themselves, the nurses, the patients, and the public. There was chaos to start on; the nurses were of indifferent status, naturally enough, because they were under-paid, and their personal and professional character was considered of secondary importance. There was no head to the staff, and it consequently lacked cohesion throughout, besides being numerically inadequate. And now comes a picture I can draw in opposition to that painted by "A Workhouse Nurse." So far from making it difficult for our nurses to work for us, we were ready within the bounds of a not too drastic advance to do anything to get well-trained and well-conducted women. We were prepared to defend them from any tyranny on the part of master and matron, and we were anxious to afford as many opportunities of recreation as were consistent with discipline and institutional service. I do not say our ideals were as high as they ought to have been, or that we even reached a standard recognised by the profession. Apparently at first we lamentably failed in both respects. Nurses came and nurses went until selections for the staff formed the staple business at the meetings of our Visiting Committee, and we all grew desperately sick of it. Sometimes we spent £20 a quarter in advertisements alone, and still we ploughed the sand. Then we decided to have a superintendent (£50) and four charge nurses (£30, rising to £36), with 14 assistant nurses (£27 to £30), two imbecile attendants (£27 to £30), and an attendant on infirm females (£20 to £25), supplanting a staff of about twelve or fourteen nurses under two charge nurses. This with an infirmary population of about 300. Soon afterwards, to our immense relief and delight, we began to see the dawn. We have now at last a permanent staff, which seems to be contented, and which promises to stay. We have not yet lost the fear of receiving resignations every time we meet, but the fear gets less terrible with every meeting that passes by without the dreaded event happening. Indeed, we almost allow ourselves to be proud of what we have achieved, though occasional echoes teach us that the wood out of which we have emerged is still not so very far off. Our nurses are capable and cheerful, and their average experience is high, and it may go almost without saying that we are satisfied with their treatment of and attitude towards our patients. We would not let them be annoyed for the place and reputation of a thousand masters and matrons, and, fortunately, in our case, there is a very satisfactory understanding between the two departments. If it came to friction and hostility, and the fault was on the side of the house—well, guardians of the old school would more than tremble for the fate of the house. Even as we stand now I am free to admit we may make a poor showing in some eyes. Possibly, our superintendent, conscious of our defects and of the small headway we have in her eyes made, has mapped out for us a magnificent progressive era for her staff, which she expects us sooner or later to obediently carry out. She may rely entirely upon our good-will, and for our part we shall only ask her to remember that we have still many prejudices to set aside, and many crochets to surmount, which a too hasty disposition of would but wreck the ultimate attainment of her paradise. Much of the work in our infirmaries is, unhappily, monotonous and dreary—the wards seem to swallow up like bottomless morasses all a nurse's skill and humanity. On that very account it appears to me we want women of unusually high calibre to withstand the depressing effects of professional life under such conditions, and I am persuaded that the more guardians of Progressive tendencies find this out the more concessions will they be willing to make to get nurses of the necessary fitness, and the more speedily will nurses be ready to enlist under boards which appreciate their difficulties, honour their self-sacrifice, and facilitate their professional advancement.

### Lectures on Medical Relief.

UNDER THE AUSPICES OF THE CHARITY ORGANISATION SOCIETY.

### DISTRICT NURSING.

MISS AMY HUGHES, Superintendent of The Nurses' Co-operation, lectured on "District Nursing" at the Portman Rooms on Friday afternoon last. Miss Hughes' long experience as Superintendent of the Central Home of the Metropolitan and National Nursing Association specially qualified her to speak of the early beginnings of that society, with a brief sketch of which the lecture began. Turning then to a general consideration of the work of district nursing, Miss Hughes pleaded for the importance of a special training for district nurses; how hopeless seemed the prospect to the hospital-trained nurse, fresh from her well-filled ward cupboards, when first introduced by her superintendent to the poor homes amongst which her new work lay! It was this very working *under* the superintendent, experienced in the special difficulties of district nursing, which was so important; the difficulties vanished when the right way to face them was pointed out. It was not only the nursing skill that was essential, but the knowledge of how to apply it, and the further qualifications of absolute courtesy and tact. The district nurse, too, whoever and whatever she might be, was also a teacher and demonstrator, and upon her enormous powers of usefulness in this respect Miss Hughes laid very great stress. The district nurse should know something of the science of ventilation, and be ready and able to overcome popular prejudices against fresh air and soap and water, knowing how to secure the former without draught, and to accomplish the necessary washing without letting the patients get chilled. It was in the power of the nurse to teach the wives and mothers many simple details in practical nursing, such as poultice and bed-making, which would be of use to them in future illness, and enable them, best of all, to help themselves. Then there was sick cookery; great prices were paid to teach cookery to the children in the Board Schools. Why might they not gain most useful experience in sick-room cookery from the district nurse, learning under her supervision how to make beef tea worthy of the name? The right woman must be got for the work, able to help on this important work of teaching. Miss Hughes then touched on the ever vexed question of the "cottage" nursing system, and strongly deprecated any lowering of the standard of district nursing in order to meet the supposed requirements of special localities. Rather should the people everywhere be raised to meet the higher standard. The difficulty was, of course, to find the means to carry out a thorough training, but if in the rush to bring help with all speed to the sick poor it was being found expedient to employ less experienced nurses, such a course would prove dangerous in the long run. Better to go more slowly and have more fully trained women. It was a serious question what would in the future become of these short-trained women; not now, whilst they were working under supervision, but presently when they were no longer thus controlled. The nurse could not fail to learn something of the resources of the family, and many were her opportunities of giving wise advice. And here Miss Hughes had a word of warning for the nurse. She must carefully guard the confidences of her poor patients; learn to hold her tongue, and eschew gossip. With regard to the question of relief, the nurse must in no sense be looked upon as an almoner. For the immediate needs of the sick she must concern herself, but that was all, and nursing societies must be careful not to overstep the rightful limits in the matter of this urgent sick relief. District nursing, carried on upon the right lines, shortened sickness, and ought to have an appreciable effect upon the expense of the community, lessening rates, as by its means many cases were kept from the workhouse.

In conclusion Miss Hughes spoke of the grants now so frequently made to district nursing societies by Boards of Guardians, and of the good work thus done; of the practical, economic value of district nursing, as a factor in binding classes together; and again of the need of supplying the poor with, if anything, even more skilled nursing than the rich, because of their greater disadvantages; and of the importance of bringing home this fact to the promoters of nursing associations.

### Minor Appointments.

HOME FOR INCURABLE CHILDREN, PARKFIELD ROAD, BRADFORD.—Miss J. M. Von Berg was appointed, on October 19th, 1898, Matron of the above. She was trained at the Seamen's Hospital, Greenwich, and her list of subsequent appointments are as follows: Sister at the Salop Infirmary, Shrewsbury; surgical sister at the Hospital for Children and Women, Bristol; temporary night superintendent at the Royal South Hants Infirmary, Southampton; and holiday matron at the Royal Victoria Hospital, Bournemouth, and at the Saltaire Hospital, Yorkshire.

BATH ROYAL UNITED HOSPITAL.—The following promotions have recently taken place at the above hospital: Nurse Mary Macdonald to be Sister of the Victoria and Cambridge wards; Nurse Maud Pidgeon to be Sister of the Helena and Katherine wards; Nurse Alice Edmonds to be Sister of the Albert and Edinbro' wards. All these nurses were trained at this institution. Amongst the probationers the gold medallist for the year is Nurse Maud Hooper, and the silver medallist Nurse Bridget Jones.

STAPLETON UNION INFIRMARY.—Miss Frances Fry has been appointed Charge Nurse of the Female Block at the above infirmary. She received her general training at the West Kent General Hospital, Maidstone. Since then has held the posts of assistant and staff nurse at the Hospital for Women, Soho Square, W., afterwards training in midwifery and obtaining her L.O.S. diploma at the Bristol General Hospital.

PEARN CONVALESCENT HOME, PLYMOUTH.—Miss M. E. Kearsey was appointed Matron here last September. She was trained at Lowestoft Hospital and at the Devon and Exeter Hospital, where she has worked since 1891, and held the post of ward sister, and has acted for the matron during her absences.

CANTERBURY PARISH WORKHOUSE INFIRMARY.—On November 15th, 1898, Miss Charlotte Annette Gilson Sheild was appointed Superintendent Nurse here. She was trained for two years at the Kent and Canterbury Hospital, and has since been attached to the Kent and Canterbury Training Institute for Nurses.

THE COTTAGE HOSPITAL, GOOLE.—On November 16th, 1898, Miss Elizabeth Pearson was appointed Matron of the above. She was trained at the Queen's Hospital, Birmingham, and was for four years matron of Whitchurch Cottage Hospital, and for three years lady superintendent of that at Fleetwood.

WESTERN GENERAL DISPENSARY, MARYLEBONE ROAD, N.W.—Miss C. E. J. Chaffer informs us that her training school was the Clinical Hospital, and not the Royal Infirmary, Manchester, as stated in the "Mirror" for November 19th.

THE COTTAGE HOSPITAL, CHIPPENHAM, WILTS.—Miss Emily Stevens has been appointed Matron of the above. She was trained at Guy's Hospital, and for the past year has been charge nurse at the Cottage Hospital, Watford, Herts.

RAINHILL COUNTY ASYLUM.—Miss Johnson's appointment to this institution, inserted in our issue of November 19th, 1898, should have been as Head Nurse and Assistant Matron, not as matron as stated.

EXETER SANATORIUM.—Miss Beaufoy has been appointed Matron here. She was trained, certificated, and held the positions of theatre sister and of ward sister at the Devon and Exeter Hospital.

### For Reading to the Sick.

ADVENT.

"THE Lord, whom ye seek, shall suddenly come to His temple."—*Malachi* iii. 1.

See the King desired for ages, by the just expected long;

Long implored, at length He hasteth, cometh with salvation strong.

Oh, how past all utterance happy, sweet, and joyful it will be,

When they who unseen have loved Him, Jesus face to face shall see!

What will be the bliss and rapture none can dream and none can tell.

There to reign among the angels, in that Heavenly Home to dwell,

To those realms, just Judge, oh, call me, deign to open that bless'd gate,

Now, whom seeking, looking, longing, I with eager hope await.

But Advent hath its double sense,

Its strain of joy and penitence,

Since He who once in mercy came

Shall come to wrap the world in flame.

Oh, spare us yet a little space

To profit by this day of grace;

Draw us by love, by mercy win,

Ere judgment come and wrath begin.

Hark, a joyful voice is thrilling,
And in each dim and ancient way
Of the ancient temple filling;
Dreams, depart, for it is day.
Christ is coming, from thy bed,
Earth-bound soul, awake and spring,
With the Sun, new-risen, to shed
Health on human suffering.
Lo! to grant a pardon free,
Comes a willing Lamb from Heaven;
Sad and tearful, hasten we,
One and all, to be forgiven.

**Reading.**

How beautiful upon the mountains are the feet of Him that bringeth good tidings, that publisheth peace; that bringeth good tidings of good, that publisheth salvation.—*Isaiah* lii. 7.

Behold the Lamb of God, which taketh away the sin of the world.—*St. John* i. 29.

Be ye also ready; for in such an hour as ye think not the Son of Man cometh.

I came down from Heaven, not to do Mine own will, but the will of Him that sent Me —*St. John* vi. 38.

The darkest hour is that which precedes the dawn, as we know full well when tossing sleepless and racked with pain on our weary sick beds. Then we pray for the light which we trust will bring us alleviation from our sufferings. Do we think, even when in November fogs and December darkness, that this darkness and gloom is merely the prelude to the lightness that will come to us at Christmastide? We can well imagine the gloom that surrounded the weary watchers nineteen centuries ago when all was dark before the coming of our Lord, and the brightness that followed when His presence was made known on earth. But the faithful were "also ready," and in God's good time our Lord came to spread brightness and light on earth. How much more should we who have heard the glad tidings be ready for the coming of the Lord? If we think of the faithful, weary of waiting in the darkness, but steadfast in their trust, should we not be ashamed of our repinings, and with glad hearts and cheerful minds prepare for the advent of our Lord?—*W. B. S.*

### Death in Our Ranks.

ON November 22nd, 1898, the nursing ranks lost Mary Elizabeth Smith, whose nursing life covered a period of fifteen years. Trained at St. Thomas's Hospital she spent four years there, and left with great regret. Another four years were passed as Sister at St. Mary's, Paddington, and abroad with a private patient. She then held the post of Head Nurse of the Isolation Block at the Sussex County Hospital, Brighton, for four more years, and finally devoted herself to private nursing. The last years of her life were spent at St. Andrew's, N.B., where she died, and to the end she was at work. Her friend Miss Bird (Woodburn, St. Andrew's, Fife) will willingly respond to any inquiry on the part of those who knew her.

### Notes and Queries.

**The contents of the Editor's Letter-box have now reached such unwieldy proportions that it has become necessary to establish a hard and fast rule regarding Answers to Correspondents. In future, all questions requiring replies will continue to be answered in this column without any fee. If an answer is required by letter, a fee of half-a-crown must be enclosed with the note containing the enquiry. We are always pleased to help our numerous correspondents to the fullest extent, and we can trust them to sympathise in the overwhelming amount of writing which makes the new rules a necessity.**

**Every communication must be accompanied by the writer's name and address, otherwise it will receive no attention.**

*Fashions in Cards*

(105) 1. Would you kindly send me the correct or fashionable way for a nurse's (ladies', certificated) card? Should I put "Nurse," "Miss," or "E. H."? 2. Also, would it be correct to put the diploma, having passed in midwifery? I have an idea that doctors do not like it; at least, I know they do not on a brass plate. Should be glad of a little advice from headquarters.—*E. H.*

1. It is a matter of taste. Go to a good stationer's, and ask to see the various styles in vogue, and choose the one you like best. 2. Certainly, put the letters denoting your certificate. No medical man will object to a card or even a brass plate inscribed "Nurse H., L.O.S."

*Antiseptics for Nurses.*

(106) Kindly inform me if "Antiseptics for Nurses," by a Medical Woman, is published in book form.—*Hope.*

Not yet. When the series of articles appearing in the "Mirror" under that title are complete the question of their republication in book form will be taken into consideration. A book entitled "Antiseptics in Midwifery" is also preparing for the Burdett Series.

*Compulsory Registration.*

(107) Kindly say if all nurses are compelled to enter their name in Burdett's Official Nursing Directory.—*S. O. N.*

S. O. N. should comply with our rules anent name and address. No nurse is compelled to enter her name in Burdett's Official Nursing Directory, but all will find it advantageous to do so.

*Fever Probationer.*

(108) Do they ever take a probationer at a fever hospital for twelve or eighteen months, and if I got into a fever hospital for that time would I be more likely to get into a general hospital than if I apply without any experience?—*E. J.*

The majority of matrons prefer probationers without previous training. Take your general training first, and your special afterwards.

*Experience in District Nursing.*

(109) Will you kindly inform me how a private nurse can get some experience in district nursing without belonging to an institution?—*A. M.*

She might board with a district nurse who had first obtained permission from her committee to receive her.

*Workhouse Infirmary Nursing Association.*

(110) Could you tell me what has become of the Workhouse Infirmary Nursing Association, late of 6, Adam Street, Strand, W.C.?—*Mildred.*

It still exists, but its work is in abeyance.

*Partially Trained.*

(111) Are there any good institutions that will train for general and monthly nursing and pay a small salary to one who has had three months' midwifery training and taken the L.O.S. in a recognised school, and who has had three months' nursing in a small hospital?—*P.*

She must begin again; but matrons, as a rule, object to partially-trained nurses. If she should find difficulty in entering a training school her only chance would be in advertising, when she might obtain work under the poor-law or in some home hospital.

*Home for Young Men in Southern France.*

We have much pleasure in acknowledging the receipt of information as to St. John's House of Rest, Mentone, from "E. E. K." and "M. B."

**ANSWERS REQUESTED.**

*Daily Nursing.*

(112) Can any of your readers tell a fully-trained and experienced nurse in what locality she could get a living simply by visiting daily? She would have to be sure of two patients a day always. Terms 13s. per week.—*Matron.*

